# Looking at Spillovers in the Mirror: Making a Case for “Behavioral Spillunders”

**DOI:** 10.3389/fpsyg.2019.01142

**Published:** 2019-05-16

**Authors:** Dario Krpan, Matteo M. Galizzi, Paul Dolan

**Affiliations:** Department of Psychological and Behavioural Science, London School of Economics and Political Science, London, United Kingdom

**Keywords:** spillover, spillunder, policy, intervention, nudging, decision-making

## Abstract

Behavioral spillovers refer to the influence that a given intervention targeting behavior 1 exerts on a subsequent, non-targeted, behavior 2, which may or may not be in the same domain (health, finance, etc.) as one another. So, a nudge to exercise more, for example, could lead people to eat more or less, or possibly even to give more or less to charity depending on the nature of the spillover. But what if spillovers also operate backward; that is, if the expectation of behavior 1 influences behavior 0 that precedes it? For example, a person may form an intention to exercise prompted by a policy intervention but overeat at present as a result. We define such a possibility as a “spillunder.” In the proposed article, we critically review the few papers that we have identified through a narrative literature review which have demonstrated spillunder effects to date, and we propose a conceptual framework. Based on evidence about the human mind and behavior from psychology and economics, we argue that spillunder effects may be more common than the limited empirical findings suggest. We propose six representative mechanisms through which the prospect of behavior 1 may impact behavior 0: executive functions, moral licensing and moral cleansing, emotion regulation, energization, construal level, and savoring and dread. We further discuss the policy and practical implications of spillunder effects and examine methodological issues that need to be considered when empirically testing these effects. As with our earlier paper on spillovers, we aim to motivate other behavioral scientists to research behavioral spillunders more systematically and extensively, and to prompt decision makers to consider these effects when designing behavioral interventions.

## Introduction

Policy makers have increasingly started adopting behavioral science insights to “nudge” behaviors ranging from energy conservation and sustainable food consumption to tax collection (Dolan et al., 2012; Ölander and Thøgersen, 2014; Halpern, 2015). Dolan and Galizzi (2015) have argued that transitioning to a second generation of behaviorally informed policy-making will require moving beyond immediate behavioral effects and investigating “behavioral spillovers” from one behavior to the next (Truelove et al., 2014; Nash et al., 2017). For example, an intervention that encourages people to donate blood may license them to subsequently display actions that are not as moral, thus donating less to environmental charities (Blanken et al., 2015).

But what if behavioral spillovers also operate backward; that is, if aiming to undertake a behavior at some point in the future influences another preceding behavior? For example, expecting to donate blood next week may license someone to behave less morally at present and discriminate a job candidate on a racial basis (Cascio and Plant, 2015). We label such a “mirror image” of behavioral spillovers as “spillunders.” This paper aims to establish behavioral spillunders as a construct that policy makers need to consider if they are to avoid unintended behavioral consequences when designing policy interventions. We start by defining spillunders, after which we propose a conceptual framework, and we overview the spillunder effects that we have identified via a narrative literature review. By examining evidence about the human mind and behavior from the field of behavioral science, we then argue that spillunder effects are likely to be more pervasive than what is suggested by the limited empirical evidence encountered through our narrative literature review. We further discuss the relevant policy implications and examine methodological considerations behind testing and measuring spillunder effects.

## Behavioral Spillunders

### Definition

**FIGURE 1:**
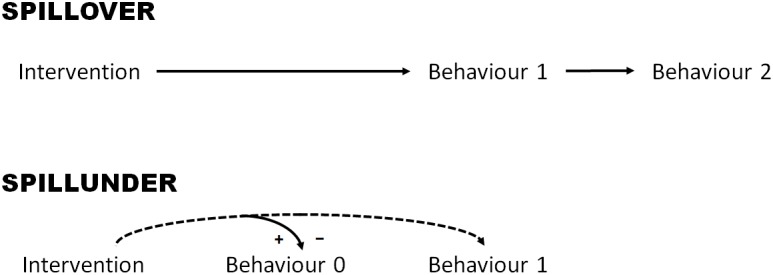
A conceptual diagram of spillover and spillunder effects. A behavioral spillover involves an “intervention—behavior 1—behavior 2” triplet, where behavior 1 and behavior 2 are two different and sequential behaviors, and the intervention is directed at behavior 1. The spillover therefore refers to the effect of the intervention on the subsequent, non-targeted behavior 2. Similarly, each spillunder involves an “intervention—behavior 0—behavior 1” triplet. As for the spillovers, the intervention is directed at targeted behavior 1. The non-targeted behavior, however, is a different behavior 0 which precedes (rather than follows) behavior 1. Behavioral spillunders thus comprise the effects that the anticipation of some behavior 1 that was induced by the intervention has on the preceding behavior 0. From this perspective, it is of a lesser importance whether or not behavior 1 ever takes place after behavior 0: what really matters is the anticipation of behavior 1 generated by different instructions about this behavior. In that sense, it is not behavior 1 itself that influences behavior 0, but the prospect of this behavior instigated by the intervention. Signs + and – refer to “enhancing” and “extinguishing” spillunders respectively, thus indicating that the prospect of some behavior 1 can either increase (+) or decrease (–) the quantity of behavior 0 or its likelihood of occurrence.

Before proposing a definition of spillunders, it is useful to recall the definition of spillovers. According to Dolan and Galizzi (2015), the starting point of any behavioral spillover is an intervention, which they broadly define as any policy intervention or experimental manipulation aimed at changing or inducing a behavior. For example, an intervention can involve a nudge that changes behavior at an automatic level, a financial incentive, a persuasion technique, an experimental instruction that informs participants to engage in certain actions, etc. Each spillover involves an “intervention—behavior 1—behavior 2” triplet, where behavior 1 and behavior 2 are two different and sequential behaviors, and where the intervention is directed at influencing the targeted behavior 1. Behavioral spillover refers to the effect of the intervention on the subsequent, non-targeted behavior 2 (Figure 1). The occurrence of a behavioral spillover is assessed experimentally by comparing the quantity of behavior 2 in a group randomly assigned to the intervention relative to a control group with no intervention (Galizzi and Whitmarsh, 2019). For example, Dolan and Galizzi (2014a) investigated how the effect of a financial incentive (intervention) on a physical activity (behavior 1)—stepping on a 6-inch high stepper—spills onto subsequent eating (behavior 2). Compared to the control condition, both high incentives (£0.10 per step) and low incentives (£0.02 per step) significantly increased the number of steps participants performed. However, whereas low incentives (vs. control) did not impact subsequent eating behavior, high incentives increased calorie intake, thus resulting in people consuming more calories than they burned.

In line with this conceptualization of spillovers, each spillunder involves an “intervention—behavior 0—behavior 1” triplet. That is, the intervention is directed at targeted behavior 1 as in the context of spillovers. The non-targeted behavior, however, is a different behavior 0 which *precedes* (rather than follows) behavior 1.^1^ Behavioral spillunder therefore refers to the impact of the intervention on this preceding behavior 0 (Figure 1). For example, in Masicampo and Baumeister (2011), all participants were told that they would need to undertake a brainstorming task that would require them to generate as many different examples of a given category as possible (behavior 1). The intervention consisted of providing participants with different instructions concerning behavior 1: in the unfulfilled goal group, they were told that they would need to list as many examples of sea creatures as they could; in the fulfilled goal group, they were given the same instructions about the sea creatures but were also asked to form a more precise plan of how they would accomplish the task (e.g., “When I get to the final task, I will write down the letters of the alphabet and will list sea creatures for each one,” Masicampo and Baumeister, 2011, p. 676); in the control condition, they were given broad instructions about having to undertake a category generation task without any reference to the specifics. Before undertaking behavior 1, all participants were administered an anagram task (behavior 0) and asked to solve as many as they could. The results showed that participants in the unfulfilled goal group solved fewer anagrams than those in the other two groups, presumably because the prospect of having to generate examples of sea creatures but without having a specific strategy that makes the task easy produced intrusive thought that clashed with the present anagram solving. As with behavioral spillovers, participants do not need to be consciously aware of what links behavior 0 and behavior 1 for spillunders to take place.

Therefore, as with spillovers, our conceptualization of spillunders naturally lends itself to imagining a longitudinal between-subject experimental setting where participants are randomly allocated to either a control or (at least) a treatment group. Participants in both groups are asked to perform exactly the same task (behavior 0). Before this task, however, they are given different sets of instructions (intervention) for a subsequent behavior 1 that will take place only after behavior 0. Behavioral spillunders thus refer to the effects that the anticipation of different behaviors 1 has on the preceding behavior 0. From this perspective, it is of a lesser importance whether or not behavior 1 ever takes place after behavior 0 (and how exactly it takes place): what really matters is the *anticipation* of behavior 1 generated by different instructions about this behavior. An alternative, but substantially corresponding, way of conceptualizing spillunders is by imagining the same longitudinal between-subject experimental setting described above, but where the intervention is behavior 1 itself: participants are randomly allocated to either the control or the treatment group; participants in both groups perform the same task (behavior 0); before performing the task for behavior 0, those in the treatment group are told that another task (behavior 1) will take place after behavior 0, whereas those in the control group are told nothing. In such a case behavioral spillunders refer to the fact that merely knowing that behavior 1 will follow can alter the preceding behavior 0.

### Conceptual Framework and Overview of the Literature

Compared to the state of research on spillovers, spillunders have been largely neglected. After conducting an extensive narrative review of the literature over the course of 2 years, we have identified only eight research articles that fall under the above definition of spillunders.^2^ Considering this limited empirical evidence, and the potentially different psychological mechanisms that account for spillover relative to spillunder effects, it would be difficult to classify spillunders using the same conceptual framework that Dolan and Galizzi (2015) developed for spillovers.

According to this framework, spillovers are divided into “promoting,” “permitting,” and “purging” (Dolan and Galizzi, 2015). Promoting spillovers refer to all behavioral sequences in which the first behavior leads to another behavior that works in the same direction. For example, if the first behavior (e.g., biking to work) is positive, which means it is consistent with an underlying motive (e.g., protecting the environment), the second behavior (e.g., recycling) is also consistent with this motive (Evans et al., 2013). Also, if the first behavior (e.g., resting) is inconsistent with a motive (e.g., losing weight) and thus has a negative sign, the second behavior follows the same direction (e.g., eating a slice of cake; Cochran and Tesser, 1996). Permitting spillovers occur when undertaking a behavior 1 (e.g., lowering water use) consistent with a motive (e.g., protecting the environment) entitles the person to undertake behavior 2 (e.g., increasing electricity consumption) that pushes back against that same motive (Tiefenbeck et al., 2013). Finally, purging spillovers occur when a person undertakes behavior 2 (e.g., donating to charity) that is driven by the motive to repair the self-image damaged by behavior 1 (e.g., being selfish; Sachdeva et al., 2009).

There are two obstacles to implementing this classification system to spillunders. First, whereas for some spillunder effects behaviors 0 and 1 are clearly linked through an underlying motive, for other spillunders a different mechanism may be at play. For example, when people expect to engage in a confrontational situation that involves warning a tenant s/he has to pay the rent (behavior 1), they are more likely to listen to music that makes them feel angry in advance (behavior 0) because this prepares them for the confrontation (Tamir and Ford, 2012). This spillunder could be categorized as promoting because the motive to collect the rent drives both behaviors to work in the same direction. In another representative spillunder research that we previously described (Masicampo and Baumeister, 2011), however, the prospect of behavior 1 may impact behavior 0 through a different mechanism: expecting to generate names of sea creatures with vs. without a plan (behavior 1) impaired anagram solving (behavior 0) due to creating intrusive thoughts rather than due to strengthening or weakening a specific motive. This spillunder, therefore, can hardly be categorized as promoting, permitting, or purging, because an underlying motive linking behavior 0 and behavior 1 may not exist, or may be difficult to identify. Another obstacle is that, even if certain spillunder effects can be classified under the categories that Dolan and Galizzi (2015) established, available evidence is not yet sufficient to support the existence of all the categories.

**Table 1:** Overview of empirical findings on behavioral spillunders.

Study	Behavior 0	Behavior 1	Finding	Spillunder Type
Masicampo and Baumeister, 2011	Solving anagrams	Generating names of sea creatures without forming a plan (unfulfilled goal) vs. generating names of sea creatures with forming a plan (fulfilled goal) vs. generating names of sea creatures with vague description of the generation task (control)	Participants expecting to generate names of sea creatures without forming a plan (unfulfilled goal group) solved fewer anagrams than those in the control group and in the fulfilled goal group	Extinguishing
Tamir and Ford, 2012	Listening to angry music clips	Confronting a tenant about paying the rent vs. nurturing a healthy relationship with the tenant (control)	Expecting to confront a tenant about paying the rent makes people more likely to listen to angry music than in the control group	Enhancing
Polivy et al., 1994	Eating cookies	Engaging in an anxiety inducing behavior (delivering a 2-min speech) vs. rating fabrics on tactile dimensions (control)	Expecting to give a 2-min speech vs. control increases cookie consumption, but only for restrained eaters	Enhancing
Morsella et al., 2010	Meditation	Generating names of all the states of the United States	Expecting to write down the names of all US states made people less able to meditate due to experiencing intrusive thoughts	Extinguishing
Urbszat et al., 2002	Eating cookies	Diet vs. no diet	Expecting to start a diet immediately after the experiment increased cookie consumption, but only for restrained eaters	Enhancing
Kopp et al., 2015	Assessment of reading comprehension of a scientific text	Focusing on behaviors that participants need to undertake after the experiment vs. removing attention from those behaviors (e.g., by making a list of the components of an automobile)	Participants who were thinking about the short-term plans they aimed to accomplish after the experiment (vs. control) performed worse on reading comprehension of a scientific text	Extinguishing
Cascio and Plant, 2015	Deciding on whether to endorse a black or a white candidate for the position of a new police officer	Engaging in a moral behavior (e.g., taking part in a fundraiser or donating blood) vs. absence of anticipated moral behavior (control)	Participants who anticipated performing a moral action in the future were more likely to reveal their racial prejudices and to discriminate a job candidate on a racial basis	Enhancing
Cody et al., 2015	Word recall task	Anticipating to undertake an anxiety inducing behavior (delivering a 5-min speech in front of the experimenter and a video camera) vs. no expectations to engage in anxiety inducing behaviors (control)	Participants with social anxiety who anticipated giving a 5-min speech falsely recalled more anxiety-related words compared to those in the control group	Enhancing


We therefore propose a simpler classification system that is appropriate for the current state of research on spillunders and can be expanded into greater detail as the number of relevant research increases. It organizes spillunders into two categories based on the direction of effect that the expectation of behavior 1 has on behavior 0: “enhancing” spillunders are those in which the prospect of behavior 1 increases the quantity of behavior 0 or its likelihood of occurrence; “extinguishing” spillunders are those in which the prospect of behavior 1 reduces the quantity of behavior 0 or its likelihood of occurrence.

Table 1 provides an overview of all the spillunder effects we identified in the behavioral science literature to date. Of the eight spillunder effects identified, three effects can be classified as extinguishing, and five as enhancing spillunders. As it can be seen from the table, the spillunder effects are spread across many different behavioral domains, including mental performance—e.g., anagram solving (Masicampo and Baumeister, 2011), reading comprehension (Kopp et al., 2015), and word recall (Cody et al., 2015); health—e.g., food consumption (Polivy et al., 1994; Urbszat et al., 2002) and meditation (Morsella et al., 2010); morality—e.g., displaying racial prejudice (Cascio and Plant, 2015); and leisure—e.g., music choice (Tamir and Ford, 2012). This variability indicates that spillunders could be relevant to many different policy domains if they are shown to be an integral component of day to day activities.

To illustrate that spillunders can be more common than the limited evidence up to date suggests, in the next section we overview a broad range of psychological and behavioral mechanisms that may account for spillunder effects across diverse situations and environments. Three of these mechanisms were selected both because they can convincingly explain some of the spillunder effects from Table 1 (as it will be discussed in section “Making the Case for Spillunders: Overview of Core Mechanisms Through Which the Prospect of Behavior 1 Could Impact Behavior 0”), and because they typically control a wide range of everyday behaviors, thus allowing us to make speculations about the occurrence of spillunders in everyday life. These mechanisms involve executive functions (plausible mechanisms that are likely behind the spillunder effects documented by Polivy et al., 1994; Morsella et al., 2010; Masicampo and Baumeister, 2011; Kopp et al., 2015); moral licensing and moral cleansing (plausible mechanisms that can explain the spillunder effects in Urbszat et al., 2002; Cascio and Plant, 2015); and emotion regulation (which can accounts for the spillunder effect in Tamir and Ford, 2012).^3^ The remaining three mechanisms—energization, construal level, and savoring and dread—were selected because of their implications for a variety of everyday actions, and because the link between the present and the future is inherently ingrained in their theorizing, thus allowing us to make convincing arguments for their involvement in spillunder effects, even if these effects have not yet been identified within their domains.

## Making the Case for Spillunders: Overview of Core Mechanisms Through Which the Prospect of Behavior 1 Could Impact Behavior 0

### Executive Functions

Executive functions refer to three broad categories of cognitive processes—inhibition, working memory, and cognitive flexibility (Diamond, 2013)—that are at the core of any behavior that is effortful and does not come spontaneously, ranging from exercise (Hagger et al., 2010) to healthy eating (Hofmann et al., 2012), and solving intellectual tasks (Mrazek et al., 2013). Inhibition comprises functions such as self-control that regulate control of attention, thoughts, behavior, or emotions, and are necessary for resisting temptations, maintaining the focus of attention, overcoming habits, and persisting on any effortful physical or intellectual tasks (Diamond, 2013). Working memory is the capacity to hold information in one’s consciousness and actively work with the information during problem solving (Engle, 2002). Cognitive flexibility allows one to look at a problem from many different perspectives, using different strategies to solve the problem, and adjusting to new situational demands and requirements to find the solution (Diamond, 2013). Without executive functions, humans would be at the mercy of their impulses and habits and would not be able to undertake any activities that require focus and effort.

Executive functions are highly susceptible to situational influences, and can be disrupted or enhanced by a variety of factors—including stress, anxiety, intrusive thoughts, cognitive load, mood, stereotype threat, mindfulness, mortality salience, and so on—which can in turn impair or enhance a variety of everyday behaviors (Sorg and Whitney, 1992; Ashcraft and Kirk, 2001; Klein and Boals, 2001; Hofmann et al., 2008, 2012; Johns et al., 2008; Stawarczyk et al., 2011; Mrazek et al., 2013). Based on this notion, it would be expected that a large proportion of spillunder effects occur via the influence of a prospective behavior 1 on executive functioning. In fact, the largest number of spillunders we identified in Table 1 can be explained by the impairment of executive functions caused by the expectation of behavior 1. For example, in Polivy et al. (1994), restrained eaters ate more cookies when anticipating an anxiety-inducing (vs. neutral) behavior because anxiety undermined their self-control, and the impulsive tendencies to indulge took over as a result. Moreover, in Kopp et al. (2015), focusing on behaviors participants were aiming to undertake after the experiment (vs. removing attention from these behaviors) likely evoked intrusive thoughts which interfered with their reading comprehension. A similar mechanism was at play in Masicampo and Baumeister (2011). Finally, in Morsella et al. (2010), intrusive thoughts activated by the prospect of having to recall the names of all US states interfered with their ability to meditate.

### Moral Licensing and Moral Cleansing

Moral licensing refers to people’s propensity to undertake an action that is less virtuous or less beneficial for their health after they have previously engaged in a morally desirable or a healthy behavior (Merritt et al., 2010). For example, purchasing an electric car may influence people to feel less obliged to act environmentally friendly compared to purchasing a conventional gas car (Klöckner et al., 2013). In contrast, moral cleansing is the propensity to engage in a morally desirable or a healthy behavior after undertaking actions that are less virtuous or healthy to restore the moral balance (West and Zhong, 2015). For example, when people think of the negative aspects of their personality or recall immoral actions from the past, they are more likely to donate money to charity (Sachdeva et al., 2009). Moral licensing and cleansing have been identified in a variety of domains ranging from pro-environmentalism (e.g., Tiefenbeck et al., 2013; Truelove et al., 2014) to health (Chiou et al., 2011), and research has established they are pervasive in everyday life (Merritt et al., 2010; Hofmann et al., 2014; Truelove et al., 2014; Blanken et al., 2015).

Moral licensing and cleansing are considered examples of “permitting” and “purging” behavioral spillovers, respectively, because their definition implies that some morally relevant behavior 1 increases or decreases the likelihood of a subsequent moral action (Dolan and Galizzi, 2015). Research has, however, shown that moral licensing and cleansing can also operate backward in accordance with our definition of spillunders. As can be seen in Table 1, the research on the influence of expecting to undertake a moral action in the future (behavior 1) on present racial discrimination (behavior 0; Cascio and Plant, 2015) is an example of a moral licensing spillunder. Another example of this spillunder from Table 1 is the impact of expected future dieting (behavior 1) on present cookie consumption (behavior 0; Urbszat et al., 2002). These findings indicate that moral licensing and cleaning may be one of the mechanisms that account for spillunder effects. Future research will need to establish whether spillunders of moral behavior are as common as their spillover counterparts.

### Emotion Regulation

Emotion regulation comprises different strategies through which people “influence which emotions they have, when they have them, and how they experience and express them” (Gross, 1998, p. 272). People regulate their emotions for a variety of reasons and in a variety of situations they encounter on a daily basis, and they generally try to regulate negative emotions more frequently than positive ones (Gross et al., 2006; Gross, 2014, 2015). Examples of emotion regulation include trying to calm oneself down when feeling angry, firing oneself up before a competitive event, or suppressing crying at a funeral (Gross and John, 2003; Gross, 2015).

There are 5 different strategies people use to regulate their emotions: (i) selection of the situation; (ii) modification of the situation; (iii) deployment of attention; (iv) change of cognitions; and (v) modulation of responses (Gross, 1998). The first two strategies are of particular interest here because of their compatibility with spillunder effects. Selection of the situation refers to “approaching or avoiding certain people, places, or objects in order to regulate emotions,” whereas modification of the situation comprises “active efforts to directly modify the situation so as to alter its emotional impact” (Gross, 1998, p. 283). An example of situation selection would be avoiding places where one is likely to meet a person one dislikes, whereas skipping a sad scene in a movie to avoid feeling negative is an example of situation modification (Aspinwall and Taylor, 1997; Gross, 2015; Livingstone and Isaacowitz, 2015).

Research showed that situational strategies of emotion regulation can result in spillunder effects: expecting to undertake some behavior 1 that can benefit from a specific emotional state may influence the person to undertake actions that potentiate that state at present. In an example outlined in Table 1, participants chose to listen to angry music (behavior 0) before confronting a tenant about paying the rent (behavior 1) because being angry makes confronting other people easier and less intimidating (Tamir and Ford, 2012). Such emotion regulation strategies in which behavior 0 is used to create or modify a situation to evoke emotions that benefit behavior 1 may be common in performance-related or confrontational contexts (e.g., sports, stock trading, or debt collection) that involve intense emotional states (Sutton, 1991; Fenton-O’Creevy et al., 2012; Lane et al., 2012).

### Energization

Energization, which is a synonym for physiological activation of the body, is typically used as an objective measure of motivation in behavioral literature and is assessed via cardiovascular reactivity indicators such as systolic blood pressure or heart rate (Brehm and Self, 1989; Wright and Kirby, 2001; Wright, 2008; Gendolla et al., 2012). Cardiovascular processes are controlled by the sympathetic and parasympathetic nervous systems and therefore encompass motivational states directed at either physical or intellectual endeavors (Obrist, 1976, 1981; Wright and Kirby, 2001; Segerstrom and Nes, 2007; Gendolla and Richter, 2010). Energization levels usually increase when people perform challenging but feasible activities (Gendolla and Richter, 2010). For example, people’s systolic blood pressure rose as the difficulty of a task that required them to memorize random letter strings increased, but systolic blood pressure dropped when the task became impossible (Richter et al., 2008). Energy levels are, however, not elevated only while people are undertaking challenging activities, but even when they anticipate engaging in such activities (Wright et al., 1986). For example, when people merely anticipated undertaking a memory task, their systolic blood pressure decreased if the task was easy compared to difficult (Contrada et al., 1984; Wright et al., 1986).

Important for spillunders, research evidence suggests that energization incited by one activity can influence behavior toward other unrelated activities, given that increased energy levels generally make a person more likely to act and more capable of pursuing demanding physical and intellectual endeavors (Wright, 2008). For example, Sevincer et al. (2014) told participants they would need to perform an intellectually demanding task (e.g., solving an IQ test) and instructed them to mentally contrast their desired performance on this task with the obstacles to achieving the desired performance level (vs. control: absence of mental contrasting). The intervention increased their systolic blood pressure. As a result, when all participants were eventually told they would not need to perform the intellectual task and were given a replacement task instead (e.g., squeezing a handgrip or writing a letter to a friend who is recovering from a car injury in the hospital), those in the mental contrasting (vs. control) condition did better on the replacement tasks.

These insights suggest that expecting to perform some effortful behavior 1 may elevate people’s energy levels and thus create various spillunder effects, depending on the context in which behavior 0 is taking place. If people are in the presence of “positive” opportunities for action (e.g., exercising or donating to charity), these spillunders may have desirable consequences (e.g., burning more calories while exercising or increased charitable donations). In contrast, if people are in the presence of negative opportunities for action (e.g., eating hedonic foods, spending electricity), the outcomes of spillunders may be undesirable (e.g., consuming more calories, increased energy use).

### Construal Level

According to construal level theory, humans can mentally represent the physical world and situations in two ways—using abstract (e.g., seeing the forest) and concrete (e.g., seeing the trees) construals (Trope and Liberman, 2010). For example, one can think of a vacation very concretely, in terms of specific activities, or abstractly, in terms of having a good time but without focusing on the details. The abstract construal is also known as high construal level, and the concrete construal as low construal level. Evidence indicates that the level of construal people use to mentally represent a stimulus (e.g., a situation or a physical object) is determined by psychological distance of the stimulus—the degree to which it is physically, socially, temporally, or probabilistically distant (Bar-Anan et al., 2006; Liberman et al., 2007; Trope and Liberman, 2010). For example, people automatically think about a place that is far away, a situation that will happen in a distant future, a person who is not close to them, or an event that has a low chance of occurring using abstract language (high construal level). In contrast, they think about a place that is nearby, a situation expected to happen soon, a person who is close to them, or an event highly likely to occur using concrete language (low construal level).

Importantly for spillunders, a high or low construal level mindset can be situationally induced and influence a variety of different behaviors: findings generally show that low (vs. high) construals potentiate impulsive behaviors by triggering present bias (Trope et al., 2007). For example, in Fujita and Han (2009) people were presented with a list of 40 words (e.g., dog) and asked to either generate exemplars of these words (e.g., poodle), which induced low construal level, or categories to which the words belong (e.g., animal), which induced high construal level. People in the state of low construal level were subsequently more likely to choose a chocolate bar over an apple. Inducing low (vs. high) construal level had a similar effect on other related behaviors such as smoking (Chiou et al., 2013), or preference for immediate over delayed outcomes (Fujita et al., 2006).

Although researchers have not yet investigated construal level theory in the context of spillunders, the research we reviewed in relation to the theory suggests that different interventions directed at behavior 1 could induce a low or high construal level mindset, thus impacting behavior 0 in accordance with this mindset. For example, if an intervention influences the person to think about behavior 1 using concrete construals, this could evoke a low construal level mindset and make the person less likely to avoid temptations at present. Moreover, if a behavior 1 is temporally close or an intervention makes it seem close, this can as well instigate a low construal level mindset and make the person more likely to act impulsively at present. Overall, each behavior 1 involves a certain element of psychological distance and can therefore incite low or high construal level (Trope and Liberman, 2010), which could in turn impact numerous behaviors relevant to health and wellbeing.

### Savoring and Dread

A small literature to date in behavioral economics has focused on accounting for instances where decision-making violates the standard assumption of positive discounting. Loewenstein (1987) is arguably the first behavioral economist to explicitly posit that the “anticipation of the future has an impact on immediate well-being” (p. 666). Earlier arguments in this direction were made by Wolf (1970), who discussed utility from memory and its implications for intertemporal choice, and by Pope (1983), who discussed the role of anticipation in risk aversion. Actually, Loewenstein (1987) traces back this same idea to Bentham (1789), for whom “anticipation, like consumption itself, was an important source of pleasure and pain” (p. 666); and to Jevons (1905), who argued that “three distinct ways are recognizable in which pleasurable or painful feelings are caused: (i) by the memory of past events; (ii) by the sensation of present events; (iii) by the anticipation of future events” (p. 3). Loewenstein (1987) shows some evidence from undergraduates (*n* = 30) who were asked their maximum willingness to pay to obtain a kiss from the movie star of their choices, or to avoid receiving a (non-lethal) 110 volts shock, with five different time delays, spanning from immediately (no delay) to 10 years in the future. Participants were willing to pay, on average, more to experience a kiss delayed by 3 days than an immediate kiss or one kiss delayed by 3 h or 1 day. The same participants were willing to pay, on average, more to avoid a shock that was delayed for 3 h to 3 days than to avoid an immediate shock. Loewenstein (1987) call “savoring” the first effect, that is the “anticipal pleasure” and positive utility derived from the anticipation of future consumption; and “dread” the second effect, that is the “anticipal pain” and negative utility derived from the contemplation of the future. Both effects cannot be explained by positive discounting, which postulates that people would prefer to consume desired outcomes as soon as possible and would prefer to delay undesirable outcomes as late as possible.

Loewenstein and Prelec (1991, 1993) relate the discussion on negative time preferences to the parallel literature on evaluating sequences of outcomes versus evaluating single outcomes. Kahneman et al. (1993), for example, found that participants strongly preferred brief sequences of decreasing discomfort even at the cost of experiencing more discomfort overall. Further evidence of preferences for improving sequences has been provided by Hsee et al. (1991) for improving sequences of relative satisfaction; Loewenstein and Sicherman (1991) for increasing 5-year salary profiles; Ross and Simonson (1991) for happy-ending experiences; Frank and Hutchens (1993) for rising wages and consumption; Loewenstein and Prelec (1993) for increasing sequences of outcomes; and Chapman (2000) for improving sequences of health outcomes. Loewenstein and Prelec (1991) observe that preferences for improving sequences can be explained in part by savoring and dread: for gains, improving sequences allow the decision maker to savor the best outcome until the end of the sequence, while for losses, getting the worst outcome immediately quickly eliminates dread. Loss aversion and adaptation can also in part explain preferences for improving sequences: over time, in fact, people tend to assimilate to ongoing stimuli and to evaluate new stimuli relative to their assimilation level so that changes in consumption, rather than levels of consumption, are the key driver of utility. While declining sequences provide a series of relative losses, improving sequences allow the decision makers to experience a continual series of positive gains from their adaptation levels. Sequences of outcomes which decline in value would thus be disliked, which indicates negative time preferences.

Frederick and Loewenstein (2008) discuss nine reasons why people may care about the profile of a sequence of events. Three reasons justify preferences for increasing sequences; three reasons justify preferences for declining sequences; and three reasons justify preferences for flat sequences which spread consumption equally across time. The three reasons for preferring improving sequences are: (i) *anticipatory utility*: delaying good outcomes extends the period over which those outcomes can be pleasurably savored, while accelerating bad outcomes reduces the period of dread; (ii) *contrast effects*: delaying consumption to future periods allows the decision makers to enjoy a series of gains relative to their “adaptation level”; (iii) *extrapolation*: people may consciously or unconsciously transform the presented sequence into corresponding longer sequences (for example, the sequence 2, 3, 4 can be preferred to 4, 3, 2 because those sequences are reinterpreted as 2, 3, 4, 5…and 4, 3, 2, 1…, respectively). The three reasons for preferring declining sequences are the same reasons for showing positive discounting and are: (i) *uncertainty* about future outcomes; (ii) *opportunity costs* from delaying outcomes which could have been profitably invested; and (iii) *pure time preferences*: genuinely caring less about utility from later periods. Finally, the three reasons for preferring flat sequences are: (i) *diminishing marginal utility* from consumption; (ii) *desire for equality among temporal “selves”* (Frederick, 2003); and (iii) “*divide-equally” heuristic*: allocating consumption among multiple periods could, consciously or unconsciously, evoke the idea of distributional equity and thus favor flat sequences (Harris and Joyce, 1980; Allison and Messick, 1990; Messick, 1993; Roch et al., 2000).

The possibility of savoring and dread is highly relevant for spillunders, since it implies that people derive utility not just from the current consumption of outcomes (behavior 0), but also from the anticipation of future outcomes (behavior 1): in many instances, the mere expectation of future outcomes (behavior 1) can affect the current behavior (behavior 0) through savoring or dread channels.

### How Widespread Are Spillunders? Summing Up “the Big Picture”

Our overview of the six widely prevalent behavioral mechanisms—executive functions, moral licensing or cleansing, emotion regulation, energization, construal level, and savoring and dread—indicates that the prospect of behavior 1 could potentially influence behavior 0 through many different routes to create spillunders. Indeed, these mechanisms shape a large proportion of everyday actions, ranging from exercise and healthy eating to pro-environmental behavior and various intellectual and moral pursuits. In fact, it would be difficult to identify more than a few activities that are not at least to some degree controlled by one or more of these mechanisms. Given the lack of research evidence on spillunders, we cannot currently determine with certainty how frequently spillunder effects occur in everyday life via these mechanisms. Our argumentation, however, suggests that even if the six overviewed mechanisms create spillunder effects in few instances, these effects may be more prevalent in day to day living than the limited evidence we identified suggests. Their under-representation in the literature therefore likely reflects the lack of effort to systematically study the phenomenon rather than its irrelevance in shaping human actions.

## From Psychological Mechanisms to Policy Implications

Spillunders have implications for any policy directed at behaviors that involve future anticipation. Whereas some policy interventions primarily concern one-off decisions that will not require any future input from the person (e.g., making decision about organ donation while acquiring the driving license; Johnson and Goldstein, 2003), other interventions affect more complex behaviors that require planning. For example, when people who have not yet paid their taxes receive a government letter that nudges them to pay the tax (Halpern, 2015), they need to decide when in the future to make the payment (e.g., on the same day, in the upcoming week, etc.). Other examples involve policies that encourage healthy lifestyle (e.g., people need to plan when to exercise or eat the healthy foods they purchased in the supermarket; Kahn et al., 2002; Story et al., 2008), or pro-environmental behavior (e.g., people need to plan time of the day when they will reduce their energy use; Schultz et al., 2007), and many others.

Any policy intervention directed at behaviors that are not undertaken immediately when the person encounters the intervention can therefore create spillunder effects. In this section, we discuss policy implications of each of the six main behavioral mechanisms that drive the impact of some anticipated behavior 1 on behavior 0. We start with moral licensing (Merritt et al., 2010). Spillunders that propagate through this mechanism are relevant to policy interventions that encourage morally responsible or healthy behaviors (Blanken et al., 2015). As can be inferred from previous research (e.g., Chiou et al., 2011; Tiefenbeck et al., 2013; Hofmann et al., 2014; Cascio and Plant, 2015), influencing people to commit to blood donation, volunteering, energy saving, healthy eating, exercising, and similar behaviors 1 in the future can backfire at present and have an undesirable impact on behaviors 0 linked to health, pro-environmentalism, charitable giving, prejudice, and so on. For example, a policy intervention that makes people more likely to plan a gym visit might also make them more likely to eat unhealthy products at present (Werle et al., 2011). To create effective policies, policy makers will therefore need to test which interventions can change behaviors 1 in the moral domain without instigating moral licensing spillunders.

Two spillunder mechanisms—executive functions (Diamond, 2013) and energization (Wright, 2008)—are relevant to any policy interventions linked to effortful behaviors that require persistence and self-control. A policy that provokes affective reactions to behavior 1 (e.g., overexcitement, anxiety, etc.) can impair executive functions and thus hinder positive behaviors 0 such as intellectual problem solving or energy saving (Hagger et al., 2010; Diamond, 2013), even if it eventually impacts the targeted behavior 1 as planned. For example, Fryer et al. (2012) showed that incentivizing teachers in advance to increase student achievement, assuming they would need to return the money if the students do not eventually improve (“loss incentive”), increased math scores compared to the traditional incentives paid upon the improved performance. Regardless of this encouraging outcome, psychology research showed that motivational strategies based on avoidance of losses can evoke anxiety and impair executive functions (Roskes et al., 2014). It is therefore a realistic possibility the loss incentive not only motivated teachers to increase student achievement (Fryer et al., 2012), but also backfired in other domains not evaluated in the experiment. In contrast to policy interventions that impair executive functions, the interventions that lead to energization—for example, by making the person committed to pursue some activating behavior 1 such as exercising or studying for school exams—can produce either desirable or undesirable spillunders, depending on which behaviors 0 the environment affords (Wright, 2008). This spillunder mechanism poses a future challenge that policy makers will need to resolve: How to build interventions that propel effortful future activities but without backfiring in a present environment regardless of the action opportunities it provides?

Construal level has implications for any policies targeting future actions because any future behavior that a person considers or anticipates can be mentally construed either concretely (low construal level) or abstractly (high construal level; Trope and Liberman, 2010). People are more likely to eventually undertake a future behavior construed concretely rather than abstractly (Liberman et al., 2007), and some of the most effective intervention strategies rely on making a targeted behavior as concrete as possible. For example, forming an implementation intention to exercise, save energy, study, or eat healthily involves formulating a plan concerning how, where, and when to undertake these activities, which eventually increases their likelihood (Gollwitzer and Sheeran, 2006; Prestwich et al., 2015). In another related line of research, participants who were shown a computer-generated older version of themselves were more likely to save for pension because the intervention made the old age more concrete (Hershfield et al., 2011). Although low construal level can be beneficial when building effective interventions that target behavior 1, it may also backfire for behavior 0, considering that concrete mind-sets increase the likelihood of acting impulsively (Fujita et al., 2006; Fujita and Han, 2009). It is therefore crucial to investigate more comprehensively how policy interventions that change construal level impact different behaviors 0 and explore how such interventions could be designed to avoid propelling impulsive present actions.

Similarly, savoring and dread imply that people derive utility not only from the current consumption of outcomes, but also from the anticipation of future outcomes. Because the overall utility at any point in time is the sum of the utility from current consumption, plus the utility from the anticipation of future consumption, it may be the case that the mere expectation of future outcomes (behavior 1) would affect the current behavior (behavior 0), for example by reducing the current consumption. Therefore, any policy which aims at influencing future behavior needs to factor in all the ramifications and the changes in the current behavior triggered by the anticipation of the future, for example in terms of savoring the future positive outcomes or dreading the future negative outcomes. This can have major consequences for the assessment of the overall impact of an envisaged policy intervention, especially if the ultimate goal of a policy intervention is the overall individual wellbeing or social welfare, rather than a narrowly defined behavioral outcomes. Given that the overall individual wellbeing is the integral over time of the instantaneous wellbeing experiences (Dolan, 2014), it is imperative that the design of behavioral interventions systematically and comprehensively capture all the spillunder effects associated to the present anticipation of future outcomes.

The final spillunder mechanism—emotion regulation—is relevant to policy contexts where the choice of some behavior 0 may be used as a strategy to propel emotional states that prepare people for behavior 1 (Tamir and Ford, 2012; Gross, 2014, 2015). For example, if behavior 1 involves using less electricity during a particular time of the day, people may undertake a behavior 0 that will make them calm and serene, so they are subsequently not tempted to engage in activities that require energy consumption. Or, if behavior 1 involves donating blood, people may undertake behaviors 0 that make them feel more powerful and less fearful, so they do not experience the act of donating blood as highly unpleasant. In this regard, the extent to which a policy directed at behavior 1 will prompt undesirable or desirable spillunders will depend on whether it propels positive or negative emotion regulation strategies. Positive emotion regulation strategies may involve activities such as mindfulness, listening to music one enjoys, socializing with friends, etc., whereas negative emotion regulation strategies may involve unhealthy eating, impulsive shopping behavior, etc. (Tugade and Fredrickson, 2007; Aldao et al., 2010; Webb et al., 2012; Gross, 2014). Understanding how to design policies that are grounded upon positive emotion regulation strategies will require researchers to dig beyond the existing knowledge on the role of emotion regulation in spillunder effects.

## Methodological Challenges

All research that has looked at behavioral spillunders so far (see Table 1) has been conducted in artificial lab settings. This may be one of the primary limitations of applying the concept of spillunder in policy making contexts: even if human behavior in the lab and in the field sometimes tend to be aligned, what happens in the lab does not always correspond to what happens in the real world (Mitchell, 2012; Alm et al., 2015; Galizzi and Navarro-Martínez, 2018). Moreover, lab experiments typically suffer from the limitation that participants know that they are part of an experiment, which in itself can alter the very behavior one is interested to investigate. An alternative is to test behavioral spillunders in “natural field experiments,” that is, in field settings where participants are not even aware that they are part of an experiment (Harrison and List, 2004). Investigating behavioral spillunders in the field, however, poses several challenges. First, whereas field experiments are designed to test the impact of an intervention on behavior 1, measuring some other preceding behavior 0 may be difficult because the experimenter does not always know whether and where the person may engage in that behavior. Second, even if the experimenter is aware where the behavior would take place, recording it may not be possible in practice.

To overcome these limitations, here we propose some suggestions for how spillunders could be measured in a more ecologically valid way to inform policy making. The first solution is to conduct “lab-field experiments” (Dolan and Galizzi, 2014b; Galizzi, 2017), that, as the name suggests, contain the elements of both lab and natural field experiments because they combine a stage where participants are observed in the lab and another stage where they are followed up over time in a natural setting while they are not aware of being observed. For example, Galizzi and Navarro-Martínez (2018) elicited social preferences in a variety of experimental games that participants completed in the lab. Participants were then invited to the lab on the next day to do a task that was not related to social preferences. After they exited the lab, they were faced with a natural field situation where they could demonstrate prosocial behavior (e.g., donating to charity, helping people), and, unbeknownst to them, their behavior was recorded.

Similar paradigms could be implemented to study spillunders. For example, imagine that one wants to investigate whether an intervention directed at physical activity (behavior 1) influences people’s donation to environmental charity (behavior 0). In that case, participants could first be invited to the lab to fill in a survey about their exercise behavior, and subsequently half of participants could receive an intervention that encourages them to behave physically active in the upcoming week (e.g., going to the gym, outdoor running). Then, after they exit the lab, all participants could encounter a natural situation where an environmental charity collects money—the amount of money donated would then be used as the dependent variable to test the spillunder effect. Additionally, researchers could also assess participants’ physical activity behavior in the upcoming week either through self-reports or through a more objective measure (e.g., Fitbit activity monitor; Takacs et al., 2014). This would allow examining not only whether intervention directed at behavior 1 impacts behavior 0, but also whether the two behaviors are eventually related.

An alternative approach could allow for the integration of behavioral science experiments with other longitudinal data. In particular, the Internet of Things (IoT; Swan, 2012) refers to the ecosystem that consists of all objects that can be connected to the Internet and generate data (Swan, 2012; Madakam et al., 2015). Some of the most obvious such objects are smartphones, laptops, and tablets, but in today’s digital age an enormous number of other objects also constitute IoT, including cars, household appliances, speakers such as Amazon Echo or Google Home, watches, etc. (Swan, 2012; Hiremath et al., 2014; Zanella et al., 2014). Almost everything can be potentially connected, and in principle people’s behavior can be continuously tracked and measured in many ways through the devices they use, their social media activities, and other online, mobile, and offline data sources (e.g., Kosinski et al., 2015).

In fact, in policy domains like health, which are typically data-rich, there is a growing interest in “behavioral data linking,” that is, in the linkage and integration of behavioral experiments with all sources of longitudinal smart data, such as hospital and electronic medical records, administrative registers, biomarkers banks, mobile devices, apps, scan data, and online panels (Galizzi et al., 2017; Galizzi and Wiesen, 2018). These same technological advances for the first time in history afford the measurement of complex behavioral patterns, such as the long-term effects or spillover and spillunder effects of behavioral interventions. For example, if all administrative records were linked together for the same individual, when policy makers send letters with different intervention messages that encourage tax payment to people (e.g., Halpern, 2015), they could potentially track the behavior of these same people in other policy contexts between the times they receive the letter and the time they submit the payment (e.g., Alzantot and Youssef, 2012; Wilson et al., 2012; Wang et al., 2014). Using this approach, it would be possible to determine which behaviors 0 participants are more likely to change as a result of the messages targeting behavior 1, as well as the direction and the magnitude of these behavioral changes.

The main obstacle to this approach is an ethical one: it is imperative to ensure that companies and organizations providing the data have obtained the general consent from participants for these data to be used for research purposes, and that the data are securely protected to avoid misuse by third parties (Sugiura et al., 2017; Baldini et al., 2018). Current developments in data protection regulation, however, such as the General Data Protection Regulation (GDPR) developed by the European Union, for example, have made the process of providing consent in such circumstances more compelling and transparent (Chassang, 2017). These and other similar developments in the legal and institutional framework may potentially increase the privacy, confidentiality, safety, and ethicality of sharing data for research purposes, and therefore enhance the potential opportunities to link online, mobile, and other longitudinal data to behavioral experiments in order to systematically investigate phenomena such as long-term effects, spillovers, and spillunders of behavioral interventions (Galizzi, 2017; Alter and Gonzalez, 2018). We therefore encourage researchers and practitioners to examine different legal, logistical, and organizational solutions and share best practices to design and implement ethically sound experiments linked with smart data when systematically testing real-world spillunder effects and their policy implications.

## Conclusion

We have proposed a definition of spillunders as the mirror image of behavioral spillovers. Spillunders are spillovers operating backward: the expectation of behavior 1 influences behavior 0 that precedes it. We have critically reviewed the few papers identified via the narrative literature review that have demonstrated spillunder effects to date and we have proposed a simple conceptual framework. Based on the evidence about moral licensing and moral cleansing, emotion regulation, energization, executive functions, construal level, and negative time preferences, we have argued that spillunder effects are likely to be more widespread than the examples that we have uncovered via our narrative literature review indicate. We have discussed their policy and practical implications. We have also examined methodological challenges that need to be considered when empirically testing for spillunder effects. As with our earlier paper on spillovers, we aim to motivate other behavioral scientists to research behavioral spillunders more systematically and extensively, and to prompt decision makers to consider these effects when designing behavioral interventions.

## Author Contributions

DK initiated and led the writing. MG contributed to the writing, in particular on the definition and methodological challenges of behavioral spillunders, and on savoring and dread. PD contributed to the writing.

## Conflict of Interest Statement

The authors declare that the research was conducted in the absence of any commercial or financial relationships that could be construed as a potential conflict of interest.

## Footnotes

1 It is important to point out that, conceptually speaking, behavior 0 can refer to various different behaviors that precede behavior 1 (e.g., behavior 0a, behavior 0b, etc.). Because we have, however, failed to identify any such more complex spillunders in the literature, we refrain from further addressing them in the present article.

2 Considering that “spillunder” is a novel conceptualization that can be used to describe a variety of different behavioral phenomena (see Table 1), we could not undertake a systematic review of the literature to detect all the articles that contain the word ‘spillunder’ effects. Our approach was therefore less systematic, and we used a variety of different strategies that led us to identify the eight research articles that involve “spillunders.” First, using Google Scholar as well as the PsycINFO, RepEC, and EconLit databases, we searched for keywords that refer to phenomena from psychology and economics under which we thought that spillunders may occur. These involve behavioral mechanisms such as energization, intrusive thoughts, planning, goal activation, goal shielding, procrastination, intentions, psychological distance, commitment, future orientation, anticipatory regret, future thinking, savoring, dread, and similar. Then we used specific keywords which refer to the link between present and future actions, such as “influence of future behavior on present behavior,” “future into present,” “future impacts present,” “future influences on present behavior,” “how future impacts present,” and similar. Finally, we explained the concept of spillunders to our close colleagues and collaborators and asked them to notify us if they encounter a paper that involves research related to this or similar constructs, and we ourselves closely followed representative psychology (e.g., Journal of Personality and Social Psychology, Psychological Science, Social Cognition, etc.) and economics (e.g., Journal of Economic Behavior and Organization, Journal of Economic Psychology, Journal of Behavioral and Experimental Economics) journals to uncover spillunder studies that may have been published in the previous issues or occurred in the most recent issues.

3 For the spillunder effect in Cody et al. (2015) (see Table 1 in the present article), we did not identify a mechanism that would convincingly explain it and could be extended to a wide range of everyday activities. This is why in Section “Making the Case for Spillunders: Overview of Core Mechanisms Through Which the Prospect of Behavior 1 Could Impact Behavior 0” we do not discuss this effect in relation to one of the mechanisms.
